# Mortality associated biological age improves independently of weight loss after bariatric surgery

**DOI:** 10.1038/s41514-026-00429-y

**Published:** 2026-06-19

**Authors:** Katharina Helena Morawitz, Wolfgang Pommer, Oleg Tsuprykov, Hendrik Krautschneider, Swantje von Germar, Nina Patzke, Felix Grassmann

**Affiliations:** 1 Institute for Clinical Research and Systems Medicine, Health and Medical University, Potsdam, Germany; 2 Charité Universitätsmedizin, Berlin, Germany; 3 Medizinisches Versorgungszentrum (MVZ), Windscheidstrasse, Berlin, Germany; 4 IFLB - Institute for Laboratory Medicine Berlin, Berlin, Germany; 5 Faculty of Medicine, Institute of Mind, Brain and Behavior, HMU Health and Medical University, Potsdam, Germany

**Keywords:** Biomarkers, Diseases, Endocrinology, Health care, Medical research, Risk factors

## Abstract

Obesity increases the risk of common diseases and mortality, placing a significant burden on our aging society. Bariatric surgery results in significant weight loss; however, the amount of associated health gain is currently less studied, particularly in the first two years. We modelled mortality-associated biological age according to established blood markers in a prospective cohort of 505 patients that underwent bariatric surgery. The difference between biological age and chronological age (age acceleration) as a molecular marker of health gain was evaluated at different time points with mixed effects models. At baseline, biological age acceleration was positively correlated to higher smoking exposure as well as increased body mass, particularly in males. Twelve months after surgery, patients were on average 5.55 years younger (slope and 95% confidence intervals (95% CI): -5.55 [-6.12; -4.97]) which remained stable until 24 months. When adjusted for changes in body mass index over time, the effect seizes decreased to 3.32 years younger age at 12 months post-surgery (slope and 95% CI: -3.32 [-4.26; -2.37]), indicating an age-rejuvenating effect – more pronounced in men - beyond weight loss. Individual markers such as glucose and C-reactive protein levels as well as blood cell counts contributing to biological age computation showed a generally more favorable change after surgery. In conclusion, biological age was markedly reduced in patients undergoing bariatric surgery resulting in a 40-50% reduction in expected mortality after two years, particularly in men. Our findings support the use of biological age as a clinically meaningful, patient-centered marker of treatment success after bariatric surgery.

## Introduction

According to the WHO, obesity is a chronic disease arising from complex interactions among genetics, neurobiological factors, eating behaviors, access to a healthy diet, market forces, and the environment^1^. In recent decades, obesity prevalence has increased globally as countries have experienced increased food security, socioeconomic development, and shifts in diet, physical activity, and societal and individual behavior due to globalization and industrialized food systems. On a global scale, the prevalence of obesity has reached epidemic proportions, with more than 1 billion individuals affected. This surge has manifested in nearly every country resulting in a consistent upward trend in obesity rates^2^ with approximately 50% of the German population having a body mass index (BMI) above 27 kg/m^2^ ^3^. Obesity has been identified as a risk factor for a variety of associated diseases, including cardiovascular diseases, type 2 diabetes, musculoskeletal disorders and several types of cancer^4^. The severity of obesity has been demonstrated to be positively correlated with an increased risk of these associated diseases.

Weight loss in obesity has been an important aspect for preventing long-term illnesses. The efficacy of a nutritionally balanced diet and increased physical activity, in reducing weight among overweight individuals is often hindered by factors that initially contributed to weight gain such as psycho-social aspects, or lack of adherence to prescribed lifestyle modifications. Bariatric surgery (BS) has been the only approved weight-loss procedure for decades^5^. It typically results in an average reduction of 20% body weight in the first year and 40% in the second year^6–8^. This weight loss translates to a reduction of all-cause mortality of about 30–50%^8^ and is thus an established treatment of weight reduction.

Traditional metrics of weight loss (e.g., changes in BMI or waist circumference or percent body weight) do not distinguish sufficiently between healthy (restorative, loss of fat mass, reduced disease risk) or unhealthy (catabolic, loss of physiological reserves, increased disease risk) weight loss. In particular, the goal of healthy weight loss is to reduce fat mass and therefore reduce future (chronic) disease risks and mortality. In comparison, unhealthy weight loss could be the results of underlying illness, frailty or excessive caloric restriction without exercise, leading to loss of physiological reserves and potentially increased disease risk. Furthermore, unhealthy weight loss could lead to false reassurance scenarios where weight loss occurs without health improvement or even worsening of disease risk^9^.

One way to define health in relation to age is to compute biological age which represents the accumulation of organ and molecular damage over time, reflecting how quickly an individual is aging compared to others. Several aging clocks used to compute biological age exist from a variety of data such as DNA methylation^10^, high-throughput *omics* data^11^ or routine blood-based markers^12^. Of particular interest are aging clocks that use markers associated with survival as they are more likely to capture mortality relevant processes and not just general senescence^13^. From those markers, the next step is to derive an aggregate score capturing the underlying liability. Mapping the score against age allows computing the biological age, effectively capturing the expected mortality risk at the individual’s chronological age. In general, it is often more informative to investigate the difference between biological and chronological age, known as age acceleration^14^, since this metric does not depend on an individual’s chronological age and thus reflects faster or slower biological aging compared to the expected trajectory^15^.

In this study, evaluated the potential of mortality-related age acceleration as an alternative marker for treatment success following BS. For this purpose, we derived phenotypic aging from blood markers at multiple perioperative time points before and assessed longitudinal changes with and without accounting for body mass index.

## Methods

### Study Cohort

The prospective investigator-initiated cohort study (BBC – Berlin Bariatric cohort Study) registered at German Clinical Trial Register (DRKS00021263) has been described previously in more detail^16^. Between December 2019 and November 2022, 1068 patients with an indication for bariatric surgery were recruited, of whom 505 underwent surgery. Participants completed detailed clinical questionnaires, and blood and urine samples were collected for laboratory analysis. Participants were followed up to 2 years post-surgery (Table 1). Follow-up data were available for 419 patients at 3 months, 386 at 6 months, 347 at 12 months, and 155 at 24 months. The median body mass index (BMI) at baseline was 44.5 kg/m² (range 30.0–78.0). The mean chronological age of the participants was 41.13 years (SD ± 11.24, range 17–69). Approximately 65% of participants reported obesity onset during adulthood.

**Table 1:** Summary statistics of the Berlin Bariatric Cohort over the period of 2 years including baseline data

Variable	Baseline	3 Months	6 Months	12 Months	24 Months
No. of participants	505	419	386	347	155
Female sex: *n*/%	384/76.00	319/76.10	302/78.20	269/77.50	120/77.40
Age: mean (S.D.) [years]	41.13 (11.24)	42.47 (11.49)	43.01 (11.28)	43.16 (11.12)	44.42 (10.88)
Weight in kg: mean (range) [kg]	132.07 (74–245)	109.81 (66–189)	96.01 (60–169)	88.11 (53–192)	87.50 (52–165)
Height: mean (range) [cm]	169.02 (148–199)	169.07 (148–199)	169.00 (148–199)	169.25 (148–199)	169.55 (151–191)
Body mass index: mean (range)	46.09 (30–78)	38.37 (25–65)	33.57 (23–55)	30.66 (20–66)	30.33 (21–49)
Therapy success^**#**^: *n*/%		137/32.70	322/83.40	326/93.90	143/92.30
Obesity onset in adulthood: *n*/%	324/65.20	273/65.20	254/65.80	230/66.30	94/60.60
High school diploma: *n*/%	125/25.20				
Non-smokers: *n*/%	370/74.4				
Surgery method					
Gastric bypass: *n*/%	152/30.100	131/31.30	134/34.70	110/31.70	58/37.40
Sleeve gastrectomy: *n* /%	353/69.900	288/68.70	252/65.30	237/68.30	97/62.60

^#^ Defined as ≥20% weight loss relative to baseline; S.D. Standard deviation

### Surgical Procedures

The two main bariatric surgical techniques were laparoscopic sleeve gastrectomy (SG) and gastric bypass (GB), performed either as Roux-en-Y gastric bypass (RYGB) or omega-loop gastric bypass (OAGB). SG involves longitudinal gastric resection, resulting in the creation of a narrow gastric tube to induce a state of reduced appetite, leading to a decrease in food intake. GB procedures combine restrictive and mild malabsorptive mechanisms by constructing a small gastric pouch and rerouting the small intestine to alter nutrient flow^5,17^. Both surgical techniques demonstrated high efficacy, with over 90% of patients achieving ≥20% weight loss relative to baseline^6^.

### Blood Biomarker Assessment

Venous blood samples were collected from participants at the time of enrollment and at scheduled follow-up visits at 3, 6, 12, and 24 months post-surgery. These blood samples were processed on the same day to measure standard hematological and biochemical parameters. From the standard panel, we extracted plasma album, creatinine, C-reactive protein, glucose, alkaline phosphatase levels as well as white blood cell and lymphocyte count, mean corpuscular volume and red cell distribution width, as shown before^12^ (Supplementary Table 1). The selected biomarkers were subsequently transformed to the appropriate scale (i.e., with appropriate units for use in subsequent analyses) and utilized to compute the phenotypic age acceleration metric (see below).

### Estimation of Mortality Risk and Phenotypic Age

The estimation of phenotypic age was conducted in accordance with the methodological framework presented elsewhere^12^. Briefly, a Gompertz proportional hazards model was used to select nine biochemical and hematological blood biomarkers (Supplementary Table 1) predictive of 10-year mortality risk. The estimated mortality risk was then mapped to chronological age thus allowing the mortality associated prediction of biological age. This approach effectively transforms biomarker-derived mortality risk into a continuous measure of biological aging expressed in years^18^ representing the expected mortality at a given chronological age. From the biological age, we derived a measure called age acceleration as a marker of faster or slower biological aging compared to actual age by computing the residual (difference) between biological and phenotypic age. Patients with a positive residual would have faster biological aging and patients with a negative residual would have slower aging than expected. Importantly, the residuals are independent (not correlated) to actual age and thus should not be influenced by differences in age at baseline and increased age during follow-up of the patients.

### Dimensionality Reduction via UMAP

Dimensionality reduction was performed using Uniform Manifold Approximation and Projection (UMAP). A non-linear embedding algorithm was designed to preserve both local and global data structures. UMAP allows to visualize relationship between individuals based on the markers supplied to identify clusters and/or identify outliers. We plotted the first two components computed from UMAP for baseline parameters with *ggplot*^19^ and color-coded with a scheme retrieved from the *RColorBrewer* package.

### Statistical Analysis and presentation of results

The correlation between biological (phenotypic) and chronological age using baseline measurements was assessed with linear regression models and the R^2^ value was extracted from the model fit.

To assess the influence of sex, baseline BMI, type of surgery, smoking status, education and the age of obesity onset on biological age at baseline, we employed multivariate linear regression models. Age acceleration was modelled as the outcome and the above-mentioned variables were jointly examined as exposures. In addition to the regression in all participants, we also fitted models with the same covariates in subgroups of patients stratified by sex, age and baseline BMI. The results of the correlation analyses are presented as correlation plots implemented in the *corrplot* package in R^20^.

To model the longitudinal change in biological age and to account for individual variability, random-intercept linear mixed-effects (LME) models were employed as implemented in the *lme4*^21^ package in R. LME models are an extension of the general linear model, incorporating both fixed effects, which represent the average relationship across all participants, and random effects, which capture deviations unique to each individual. We allowed for random intercepts for each individual by including the study ID as random effect and adjusted all models for sex. In a sensitivity analysis, we also included BMI measured at each time point to account for changes in BMI during follow-up. The changes in biological age at different time points are shown using a *beeswarm* plot, as implemented in the *beeswarm* library in R^22^. To illustrate the longitudinal trajectory in biological age while accounting for changes in the BMI of individuals, we extracted the residuals from a linear regression model with age acceleration as the outcome and BMI as the sole predictor. The residuals represent the BMI-adjusted age acceleration (changes in biological age) and were subsequently also visualized using *beeswarm* plots. In all analyses, *p*-values below 0.05 were deemed as statistically significant and highlighted with an asterisk in the respective correlation and *beeswarm* plots.

## Results

### Multidimensional analysis of mortality-associated biomarkers

**Fig. 1:**
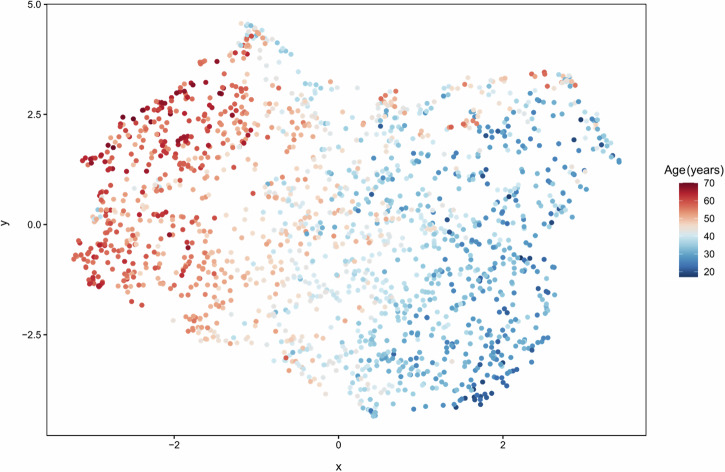
Cluster analysis of 505 obese patients according to mortality-associated biomarkers at baseline. Uniform Manifold Approximation and Projection (UMAP) was used to map the 9 mortality associated biomarkers to two dimensions for individual patients. Each point represents a single participant, colored according to chronological age (blue: younger, red: older). Distinct clustering patterns by age are observed, with a continuous gradient from younger to older individuals across the UMAP space.

Mapping nine mortality-associated biomarkers to two parameters with UMAP revealed a continuous, age-ordered distribution of individuals across the two-dimensional manifold (Fig. 1). Participants were arranged along a gradual trajectory from younger to older age, without the presence of distinct, separable clusters. This finding indicates a progressive shift in biomarker composition across the aging continuum as expected from age- and mortality-associated markers.

### Association between chronological age and phenotypic age

**Fig. 2:**
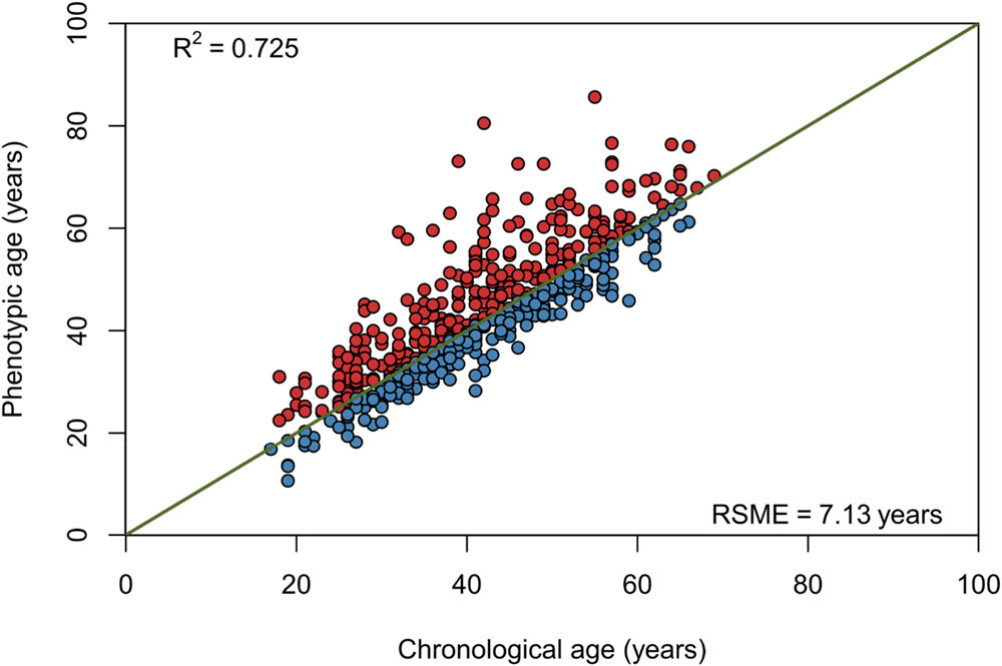
Relationship between chronological and biological (phenotypic) age in a high BMI cohort. Scatter plot depicting the correlation between chronological age and phenotypic age at baseline. Red points indicate individuals with a higher phenotypic age than chronological age (accelerated biological aging), blue points indicate individuals with a lower phenotypic age (slower biological aging). R^2^ = coefficient of determination; RMSE = root mean square error.

Phenotypic age, a composite biomarker-based estimate of biological aging, exhibited a strong linear relationship with chronological age at baseline (R² = 0.725; RMSE = 7.13 years) (Fig. 2). Similar correlations were observed in male and female patients (men R² = 0.739, women R² = 0.720). The distribution of data points indicates a considerable inter-individual variability in the difference between phenotypic and chronological age. This deviation, termed “age acceleration” (AgeAccel), is defined as the residual of phenotypic age when chronological age is regressed out. This approach is equivalent to computing the difference between predicted (biological) age and actual (chronological) age. Individuals with higher-than-expected phenotypic age (faster biological aging) demonstrated positive AgeAccel values, while those with lower-than-expected phenotypic age demonstrated negative AgeAccel values (Fig. 2).

Generally, AgeAccel was not correlated to chronological age at baseline (*p* > 0.05), neither in individuals younger nor older than the median. Thus, heteroscedasticity is unlikely to influence our results, and we opted not to adjust our models for age at baseline or follow-up. To put our results into perspective, we correlated age acceleration against the mortality risk score at baseline and estimated that a one-year reduction in age acceleration results in a relative reduction of the mortality risk by about 8%.

### Correlation between AgeAccel and demographic/clinical parameters at baseline

**Fig. 3:**
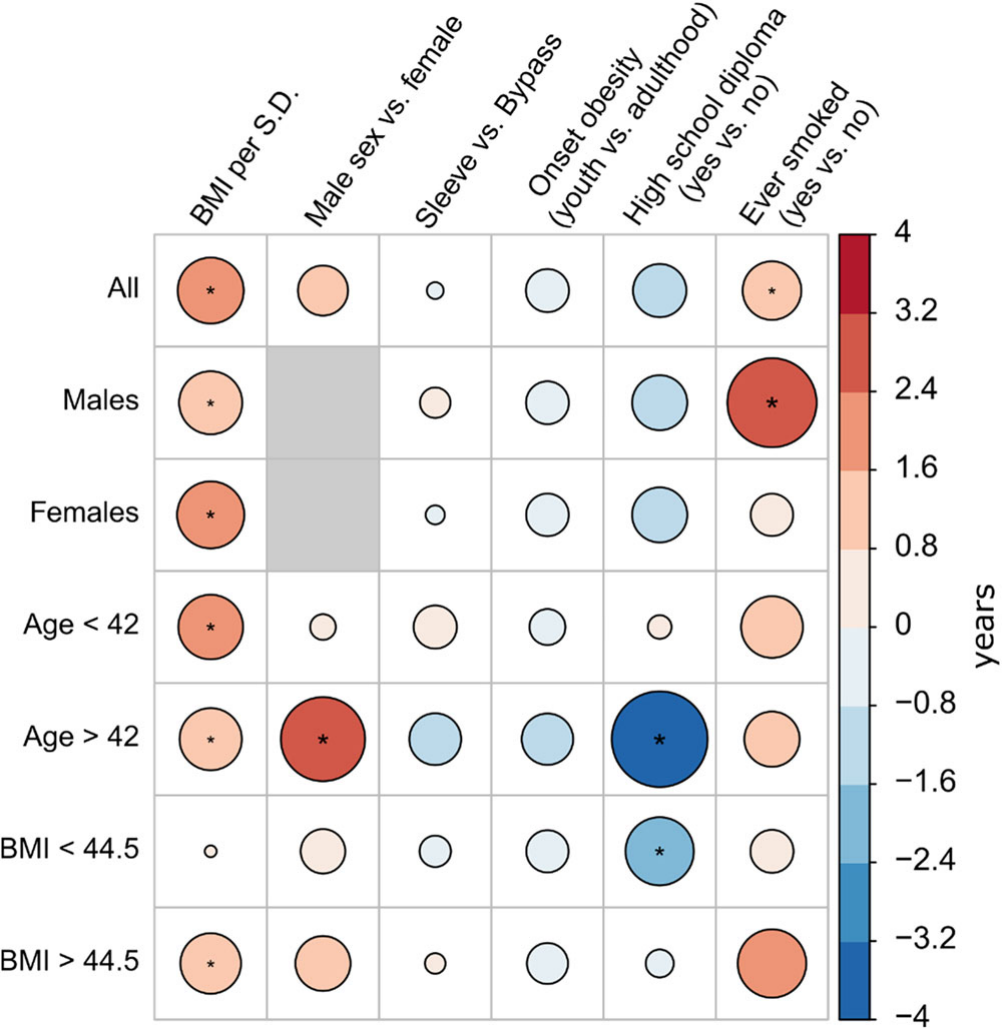
Associations between demographic/clinical variables and Age Acceleration. Shown are the correlations between clinical and demographic factors and age acceleration before surgery across participant subgroups. The color scale denotes the direction of correlations (blue: negative, i.e., younger biological age, red: positive, i.e., older biological age), and bubble size reflects the strength of the association in years. Higher body mass index and smoking were both correlated to faster aging. S.D. = Standard deviation.

Correlation analyses conducted across the entire cohort demonstrated a significant positive correlation between AgeAccel and BMI (slope and 95% confidence intervals (95% CI) per standard deviation of BMI: 1.71 years [1.13; 2.3], p = 1.59*10^-8^, Fig. 3). This correlation was also significant in all strata analysis except in the lower BMI group (BMI < 44.5: slope per S.D. and 95% CI: 0.05 years [-0.70; 0.81], *p* = 0.88).

We found that over all age groups, women were not biologically older or younger than men. However, in individuals over the median age of 42, we found that women are indeed biologically younger than men (slope and 95% CI: -2.78 years [-4.75; -0.81], p = 0.006) (Fig. 3). In this age group as well as in the lower BMI group, education level (Highschool diploma; yes vs. no) showed a significant correlation to AgeAccel with higher education resulting in reduced age (slope and 95% CI: -3.6 years [-5.71; -1.48], *p* = 0.001 and -1.83 years [-3.56; -0.1], *p* = 0.04, in the younger age group and lower BMI group, respectively). In addition, we observed that smokers had advanced age compared to non-smokers (slope and 95% CI: 1.35 years [0.04; 2.65], *p* = 0.04). This correlation was more pronounced in male smokers compared to male non-smokers (slope and 95% CI: 3.12 years [0.38; 5.86], *p* = 0.028).

### Longitudinal changes in biological age after bariatric surgery

**Fig. 4:**
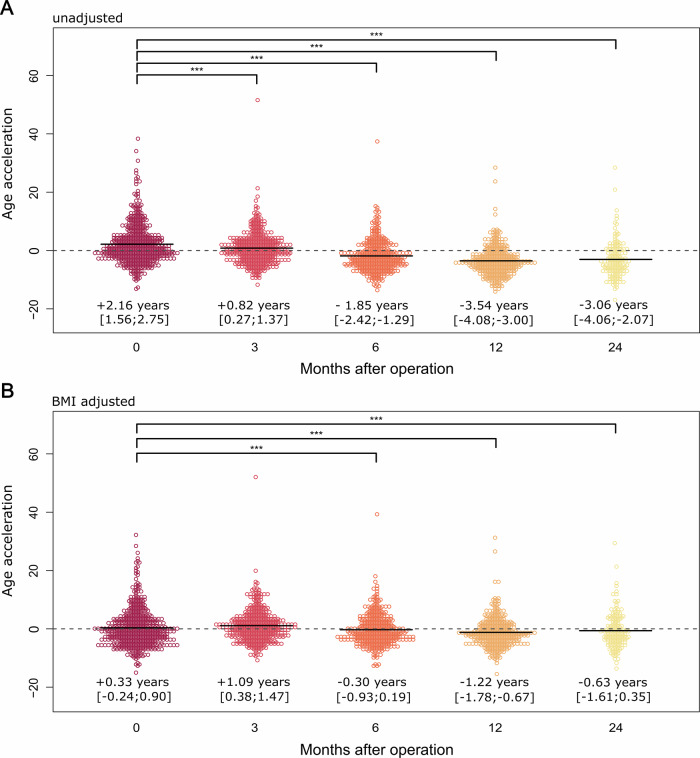
Longitudinal changes in biological age following bariatric surgery. Beeswarm plots showing biological age acceleration at baseline and at follow-up after bariatric surgery (BS). Each point represents one individual. Below each plot, the median and 95% confidence intervals are given. **A** A significant reduction in biological age acceleration is observed at 3, 6, 12 and 24 months post-surgery (mixed effects models adjusted for sex). **B** After adjustment for BMI, the reduction in biological age at 6, 12 and 24 months post-surgery (mixed effects models adjusted for sex and BMI) remains significant. At 3 months post-surgery, biological age acceleration is slightly elevated, suggesting a transient rise in mortality risk in the early postoperative period, *** p < 0.0001.

Employing mixed-effects models revealed significant postoperative changes in AgeAccel across four follow-up visits at 3, 6, 12, and 24 months after surgery (Fig. 4). We found that biological age decreases significantly (Fig. 4A) at 3, 6, 12 and 24 months after surgery by 1.34 years (slope and 95% CI: -1.34 [-1.87; -0.80], *p* = 1.17*10^-6^), 4.00 years (slope and 95% CI: -4.00 [-4.56; -3.44], *p* = 8.33 *10^-42^), 5.55 years (slope and 95% CI: -5.55 [-6.12; -4.97], *p* = 1.32*10^-70^) and 5.27 years (slope and 95% CI: -5.27 [-6.06; -4.49], *p* = 2.70*10^-37^), respectively. Those estimates would thus result in a reduction of around 40–50% in all-cause mortality risk 12 and 24 months post-surgery.

Since patients lost weight during follow-up, this effect can potentially be explained by reduced BMI instead (since we showed that BMI is correlated to AgeAccel in Baseline). To test this, we adjusted the mixed effects models by BMI measured at each time point (Fig. 4B). Similar to the results obtained without accounting for BMI, we found that biological age significantly decreased at 6, 12 and 24 months post-surgery by 2.18 years (slope and 95% CI: -2.18 [-3.01; -1.36], *p* = 2.33 *10^-7^), 3.32 years (slope and 95% CI: -3.32 [-4.26; -2.37], *p* = 7.94 *10^-12^) and 2.93 years (slope and 95% CI: -2.93 [-4.04; -1.82], *p* = 2.63 *10^-7^), respectively (Fig. 4B). However, the decrease was not as pronounced as observed in the unadjusted analysis, indicating that weight loss accounted for around 40–50% of the reduction in biological age.

In the early postoperative period (i.e., 3 months post-surgery), biological age was slightly increased by 0.19 years (slope and 95% CI: 0.19 [-0.85; 0.47], *p* = 0.57), however did not reach a statistical significance compared to baseline on average when accounting for BMI.

**Fig. 5:**
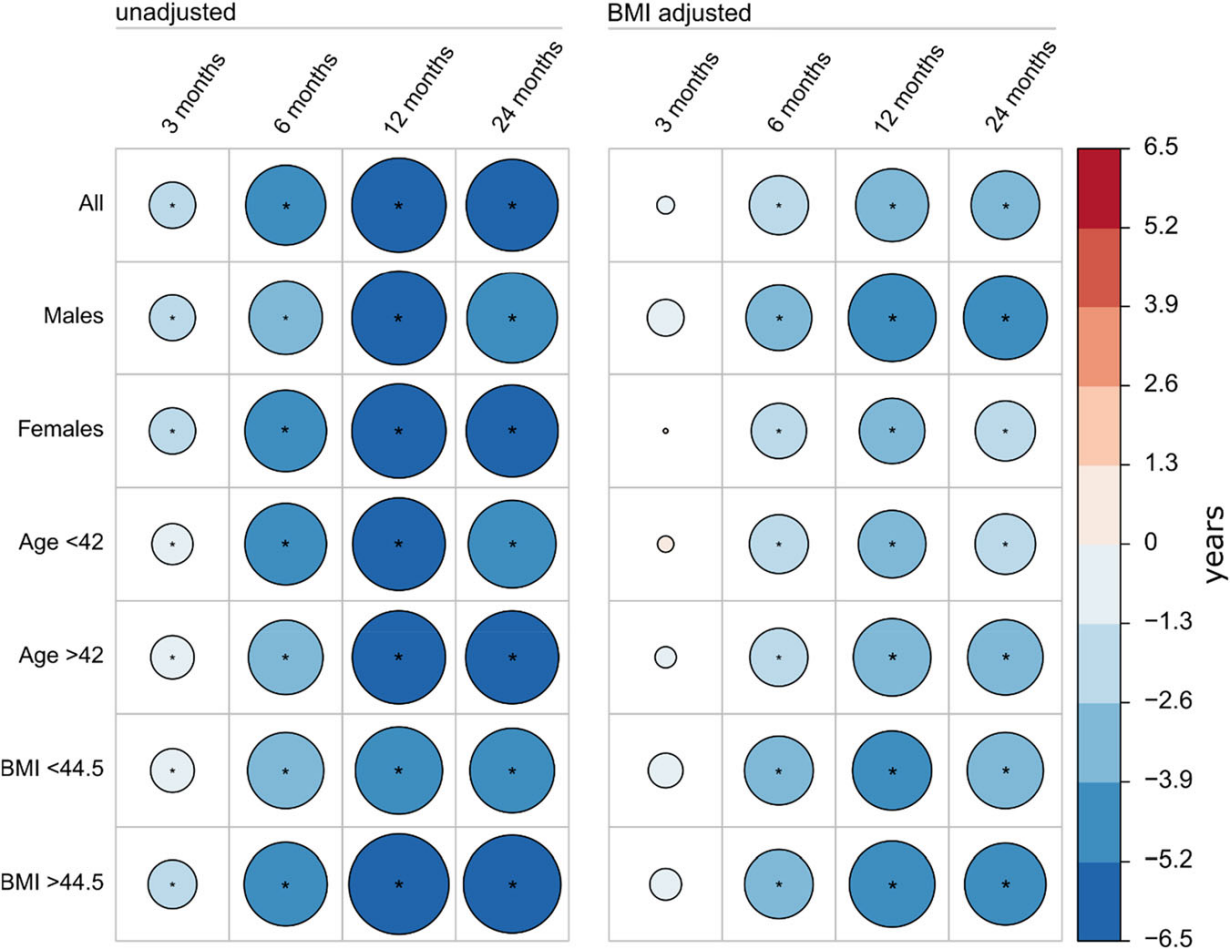
Longitudinal changes in biological age across time points and subgroups. Correlation plots depict correlations between follow-up time points (3, 6, 12, and 24 months post-surgery) compared to baseline and age acceleration across participant subgroups, with and without adjustment for BMI. Color represents the direction and magnitude of correlations (blue: negative, i.e., younger biological age, red: positive/older biological age), while circle size indicates effect strength in years. Asterisks denote statistical significance at *p* < 0.05. Across all participants, age acceleration showed a progressive negative association with time, reaching the strongest effect at 24 months post-surgery. Adjustment for changes in BMI attenuated the observed correlations by about 50%.

To see whether those observed effects are influenced by biometric or demographic baseline variables, we fit the same models stratified by sex, age and pre-surgery BMI (Fig. 5). Generally, the correlation pattern was similar to the overall analysis including all patients. Nevertheless, we observed a difference in the reduction of AgeAccel during follow-up when accounting for BMI at 12 and 24 months depending on patient sex. We found that biological age decreased more in men at 12 months post-surgery (slope and 95% CI: -4.68 years [-6.59; -2.76], *p* = 2.39*10^-6^) compared to women (slope and 95% CI: -2.50 years [-3.65; -1.36] *p* = 1.86*10^-5^). This difference remained stable also at 24 months post-surgery. Since the 95% confidence intervals overlap between the estimates and men and women, we did not find a statistically significant difference between the effect sizes, which can be attributed to the limited sample size of male patients in our cohort.

### Longitudinal changes in biological markers of biological age after surgery

Generally, most individual markers used to compute biological age (Supplementary Table 1) were significantly changed 3 months after surgery and thereafter (Supplementary Figure 1). Indeed, we observed reduced levels of glucose, creatinine, alkaline phosphatase, C-reactive protein, and white blood cell count, indicating improved metabolic, renal and liver health as well as reduced systemic inflammation. Most markers stayed relatively stable over the rest of the follow-up period. Notably, C-reactive protein and alkaline phosphatase levels continued to decline until 12 months post-surgery, indicating further improvements after the early postoperative period (3 months).

When accounting for the rapid weight loss (and thus BMI), we found that the trajectory of most markers is attenuated and showed weaker correlations with the exception of glucose and creatine, which showed a correlation pattern similar to the unadjusted approach and red cell distribution width (RDW), which exhibited slightly higher correlations at three months post-surgery. While weaker, we still found that C-reactive proteins levels decreased, and lymphocyte levels increased after 6 months post-surgery compared to baseline (Supplementary Fig. 1).

## Discussion

In this study, we examined the change in biological age related to overall mortality after bariatric surgery (BS). We could demonstrate for the first time an average reduction of biological age by about 6 years at 12 and 24 months after BS resulting in a strongly decreased mortality. The reduction in age acceleration was attenuated by adjusting for changes in body mass index (BMI) across the follow-up, indicating that most of the rejuvenating effect can be attributed to weight loss. Sex-stratified analyses indicated stronger decreases in males compared to females. Importantly, we saw no biological age rejuvenation at 3 months post-surgery when accounting for BMI reduction at this time point.

Our analysis yielded a R² of 0.725 when investigating the correlation between chronological and predicted (phenotypic) age, which is lower than the correlations reported in prior studies (R² ≈ 0.85)^23^. This can be due to cohort-specific characteristics, such as the higher baseline BMI. We note that a notable gap in the research exists concerning the influence of extreme adiposity on biological aging metrics. To date, the number of studies that have examined BMI as a modifying factor in biological age assessment is limited and often relied on self-reported retrospective BMI ascertainment or was only conducted in a cross-sectional setting^18^. In addition, in our cohort, BMI varied greatly among participants. Thus, predicting age from blood markers could yield a lower R^2^ value due to this added (and generally understudied) variation.

The observed reduction in biological age after surgery was accompanied by a decline in estimated mortality risk of about 40–50% between 6 and 24 months post-surgery compared to baseline. This observation aligns with the findings from studies demonstrating that BS reduces all-cause mortality and improves long-term cardiovascular and metabolic outcomes^5,7^ at comparable magnitudes. The most significant decreases in biological age were revealed at 12 and 24 months post-surgery. This time frame also corresponds to the period of optimal metabolic normalization following BS^24^, which might be the driving factor behind our findings in addition to the reduction of fat mass during the same period.

Across the entire cohort, biological age showed a decline over time following BS. Crucially, we observed that after accounting for BMI, we detected differences in the change of age acceleration after surgery according to sex. Indeed, male subjects showed a more substantial decrease in biological age at 6,12, and 24 months post-surgery compared to women. These findings suggest that biological age reduction following BS may be more responsive in men than in women. This might potentially be explained by our observation that male subjects entered into the intervention with a higher baseline biological age and thus potentially more disease burden. The results of other epidemiological studies support our observations: male patients exhibited a higher prevalence of comorbidities prior to surgery and consequently initiated treatment from a disadvantageous position^25^ and could thus have a greater potential for post-surgical improvement.

In addition, some studies found a more substantial reduction in fat mass in males compared to females. However, our study did not ascertain fat mass at baseline or beyond, thus we cannot investigate such a link nor control for it in the statistical analyses. In longitudinal studies, there were no observed differences between sexes regarding overall survival^26–28^. Importantly, those studies compared average survival between treated and untreated patients in the gender strata, thus could miss individual effects that only appear when investigating markers at multiple time points in the same individual, thus reducing the inter-individual variation. As indicated by the findings of other studies, BMI of men continues to exceed that of women one year post-surgery despite larger weight loss, which could explain why we found differences between sexes when investigating absolute changes in biological age and thus mortality compared to relative changes as prior studies did^27^. In addition to those explanations, stronger alterations in behavioral factors in men compared to women, such as differential adoption of lifestyle modifications, physical activity, or dietary changes, were not captured in this study and may also contribute to the observed gender differences. Alternatively, biological age may be more sensitive to weight reduction in men than in women. However, given the smaller number of male participants, these interpretations should be considered hypothesis-generating rather than definitive.

At current, we can only speculate which pathways are responsible for the slightly increased biological age observed at three months when accounting for body mass index, particularly in female participants. This finding can be due to acute postoperative stress responses, including inflammation, immune activation, and wound-healing processes. These processes are known to influence leukocyte-derived biomarkers that are incorporated into biological age calculations. Concurrent elevations in inflammatory and stress-related pathways have been documented in early postoperative bariatric cohorts^29^. Due to the immune modulatory nature of estrogen, a higher response of those markers in women is possible and could explain some of the observed sex differences. In addition, early postoperative food restriction defined as a state of negative energy balance and mobilization of endogenous energy stores may further contribute to this effect by initiating responses similar to those associated with starvation. The latter finding could be explained by the increased red cell distribution width (RDW) observed at 3 months post-surgery, even when accounting for BMI reduction at this time point. Indeed, prior reports found that malnourishing can result in increased RDW^30^, pointing towards an imbalance of nutrition at this stage.

Beyond its descriptive value, biological age modeling has important translational implications. The method outlined in this paper could be applied to calculate the health effects of various obesity interventions, including drug treatment (such as GLP-1-receptor agonists) and lifestyle modifications to monitor treatment success and to inform the necessity of additional interventions in case an adequate reduction in biological age is not achieved. In addition, future studies should focus on the impact of weight regain after bariatric surgery and other interventions on biological age. Previous epidemiological studies indicated that moderate weight regain does not translate to an, on average, increased mortality risk and only minor increase in cardiovascular risk^31^. Nevertheless, biological age might offer a more granular evaluation of disease risk after weight regain, since some patients might show resilience against weight gain (i.e., no increased biological age despite weight increase) while other might suffer from a more pronounced negative impact on their biological age and thus mortality risk. Those patients might profit from additional interventions to counter the increased risk. Thus, knowledge on health gain resilience might be crucial to improve health in individuals after weight loss. The application of blood-based biomarkers facilitates scalable, minimally invasive estimation of biological age and mortality risk at the individual level across multiple visits. This approach goes beyond abstract percentage-based descriptions of weight loss and provides patients with tangible, personalized indicators of health improvement. Given the heterogeneity of treatment success in BS and the limitations of weight-based metrics in fully capturing this heterogeneity, biological age may serve as a complementary endpoint to assess individualized therapeutic benefit.

A significant strength of this study is the ongoing, prospective data collection within the BBC, which allows the longitudinal evaluation of biological age. The notable number of female participants in this study addresses a significant gap in obesity research, which has historically been male-biased. Furthermore, the cohort spans a wide BMI spectrum (30–78 kg/m²), thereby enabling the assessment of biological aging across levels of adiposity that remain underrepresented in current research. By also evaluating anamnestic markers in this obese cohort allowed us to further shed light on the contribution of those factors to biological age, which seem to be of limited impact in those individuals.

However, we also note that BMI is a suboptimal metric for evaluating adiposity and metabolic health. Since current guidelines often define a successful BS as achieving at least 20% weight loss from baseline, we nevertheless opted to use BMI as an important factor in our analyses to monitor weight changes after baseline. Furthermore, the study had a higher attrition rate due to the lack of required follow-ups after surgery leading to fewer observations after 12 and 24 months, which could limit our power to draw further conclusions from the data.

## Conclusion

The present study demonstrates a substantial reduction in biological age and thus mortality risk after bariatric surgery, with particularly notable effects in the male population. Importantly, we found that a part of the reduction in biological age was independent of BMI changes, suggesting that bariatric surgery exerts systemic benefits beyond weight loss itself. These findings support the use of biological age as a clinically meaningful, patient-centered marker of treatment success that is less abstract and potentially more informative than weight-based metrics alone or metrics that focus solely on beneficial changes in future disease risk.

### Ethical standards

The study was approved by the ethical review board of the Ärztekammer Berlin (Eth-15/19, registration date 2020-04-15). Informed consent was obtained from all individual participants included in the study.

## Supplementary information

Supplementary Information

## Data Availability

The analyses script can be shared upon reasonable request. Data underlying the results cannot be shared due to ethical constraints.
