# Supplementary material for: Mortality associated biological age improves independently of weight loss after bariatric surgery
Source: NPJ Aging. 2026 Jun 19;12(1):86. doi: 10.1038/s41514-026-00429-y (PMC13280343; doi:10.1038/s41514-026-00429-y)
Supplement: Supplementary file 1 — Supplementary Information [file 41514_2026_429_MOESM1_ESM.docx]

## **Supplementary Figures and Tables**


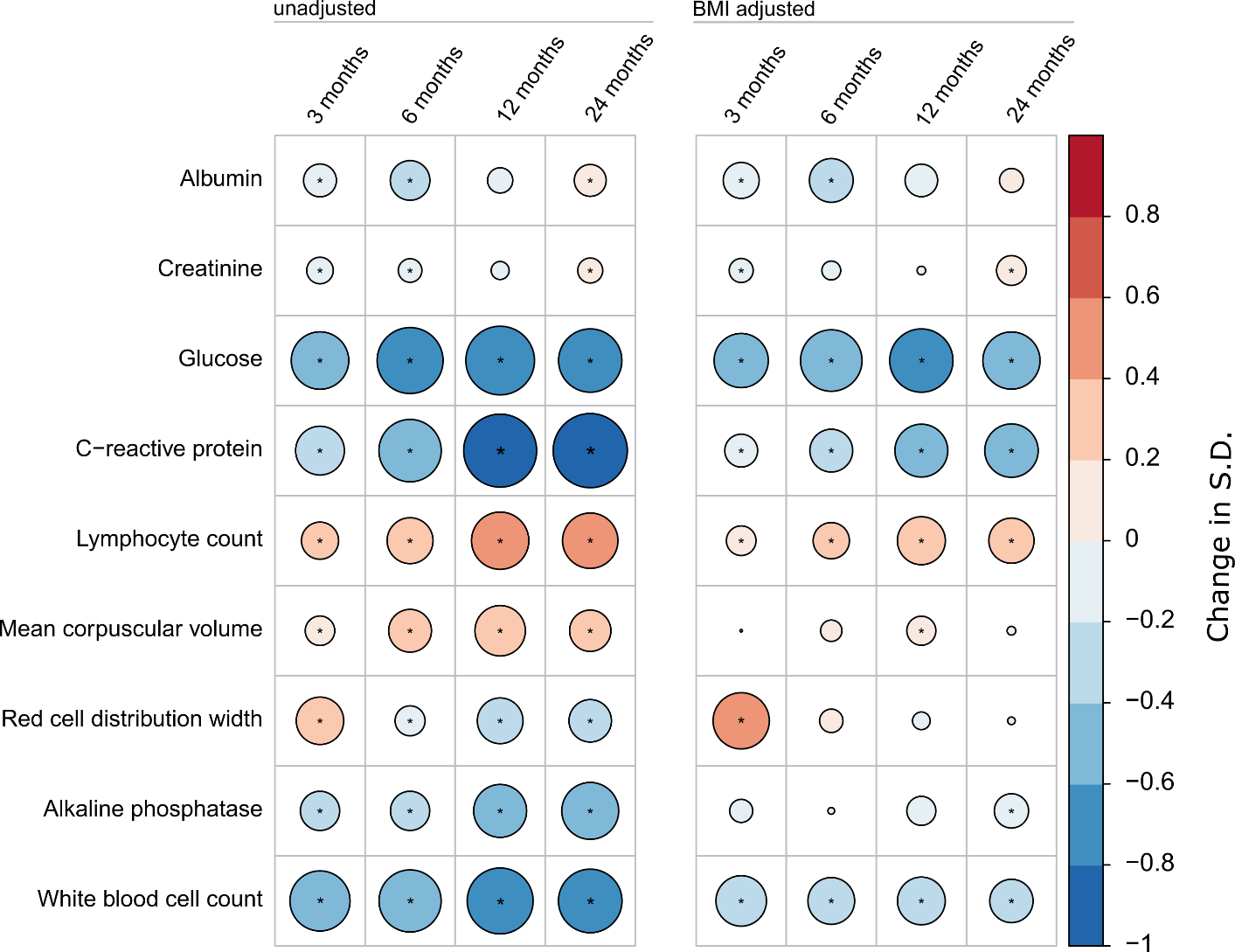


**Supplementary Figure 1. Longitudinal changes in blood markers used for biological age calculation after bariatric surgery.** Correlation plots depict correlations between follow-up timepoints (3, 6, 12, and 24 months post-surgery) and blood markers, before and after adjustment for covariates. Color represents the direction and magnitude of correlations (blue: negative, i.e., reduction of the marker compared to baseline, red: positive/increased marker levels), while circle size indicates the effect strength as standard deviations (S.D.) from the mean of each marker. Asterisks denote statistical significance at p < 0.05.

| Variable | Baseline | 3 Months | 6 Months | 12 Months | 24 Months |
| --- | --- | --- | --- | --- | --- |
| Albumin mean (S.D.) [g/L] | 41.64 (2.56) | 41.19 (2.40) | 40.83 (2.77) | 41.36 (3.22) | 41.69 (3.00) |
| Creatinine mean (S.D.) [µmol/L] | 62.95 (18.16) | 61.23 (25.06) | 61.22 (22.24) | 62.27 (19.92) | 62.73 (12.59) |
| Glucose mean (S.D.) [mmol/L] | 5.61 (1.80) | 4.99 (1.06) | 4.76 (0.86) | 4.71 (0.78) | 4.91 (1.14) |
| C-reactive protein mean (S.D.) [mg/dL] | 1.12 (0.97) | 0.80 (1.07) | 0.57 (0.76) | 0.36 (0.65) | 0.35 (0.37) |
| Lymphocyte count mean (S.D.) [%] | 28.61 (7.03) | 30.03 (7.05) | 30.99 (7.54) | 32.56 (7.81) | 31.49 (8.15) |
| Mean corpuscular volume mean (S.D.) [fL] | 85.60 (5.29) | 86.49 (4.49) | 87.04 (5.17) | 87.91 (4.65) | 87.64 (5.14) |
| Red cell distribution width mean (S.D.) [%] | 14.12 (1.28) | 14.52 (1.14) | 14.00 (1.08) | 13.72 (1.00) | 13.75 (1.25) |
| Alkaline phosphatase mean (S.D.) [U/L] | 84.89 (21.37) | 80.05 (21.91) | 80.06 (22.27) | 76.03 (23.96) | 78.02 (22.59) |
| White blood cell count mean (S.D.) [1000 cells/µL] | 8.14 (2.33) | 6.98 (1.97) | 6.82 (1.89) | 6.66 (1.90) | 6.85 (1.95) |

**Supplementary table 1: Summary statistics of the blood markers used for phenotypic age calculation**

S.D. = Standard deviation
